# Inhibitory effect and related mechanism of decitabine combined with gemcitabine on proliferation of NK/T cell lymphoma cells

**DOI:** 10.3389/fphar.2023.1134895

**Published:** 2023-03-01

**Authors:** Lanke Lin, Xiangqin Liu, Hui Yu, Huan Deng, Kun Peng, Jiang Chen, Chunle Zhang, Tao Jiang, Xiaoqi Liu

**Affiliations:** ^1^ College of Medical Technology, Chengdu University of Traditional Chinese Medicine, Chengdu, China; ^2^ Department of Laboratory Medicine and Sichuan Provincial Key Laboratory for Human Disease Gene Study, Sichuan Provincial People’s Hospital, University of Electronic Science and Technology of China, Chengdu, China; ^3^ Department of Laboratory Medicine, The People’s Hospital of Leshan, Leshan, China; ^4^ Health Management Center, Sichuan Provincial People’s Hospital, University of Electronic Science and Technology of China, Chengdu, China; ^5^ The Department of Ophthalmology, Sichuan Provincial People’s Hospital, University of Electronic Science and Technology of China, Chengdu, China; ^6^ Division of Nephrology, Kidney Research Institute, West China Hospital of Sichuan University, Chengdu, China; ^7^ Department of Hematology, Sichuan Academy of Medical Sciences & Sichuan Provincial People’s Hospital, Chengdu, China; ^8^ Sichuan Provincial Key Laboratory for Human Disease Gene Study, Center for Medical Genetics, Sichuan Academy of Medical Sciences & Sichuan Provincial People’s Hospital, University of Electronic Science and Technology of China, Chengdu, China; ^9^ Research Unit for Blindness Prevention of Chinese Academy of Medical Sciences (2019RU026), Sichuan Academy of Medical Sciences & Sichuan Provincial People’s Hospital,, Chengdu, Sichuan, China

**Keywords:** NK/T cell lymphoma, decitabine, gemcitabine, ferroptosis, apoptosis, drug combination

## Abstract

**Background:** EBV-associated lymphoma is a neoplasm with a poor prognosis, highly aggressive, and progressive rapidly. There is no standard clinical treatment protocol. Decitabine and gemcitabine are known to have anticancer properties against cells of various cancer, respectively. However, the effect of the combination medication on NK/T cell lymphoma cells and potential mechanisms have not been thoroughly investigated.

**Methods:** Human NK/T cell lymphoma cells NK92MI were treated with decitabine and gemcitabine alone or in combination. Experiments, including the Cell Counting Kit-8 and flow cytometry, were performed to investigate how the combination of decitabine and gemcitabine affects the biological behavior of NK92MI cells *in vitro*. mRNA sequencing, RT-PCR, and western blotting were used to detect changes in the related signal pathway, mRNA, and protein expressions.

**Results:** Decitabine and gemcitabine significantly inhibited the viability and proliferation of NK92MI cells in a dose-dependent manner. The combination index was less than 1 after treating with two drugs, which was a significant synergistic effect. The decitabine concentration with the best synergistic effect was 4.046 µM, and the gemcitabine concentration was 0.005 µM. Flow cytometry showed that combining two drugs could significantly promote apoptosis and arrest the cell cycle at the S phase. In the combined DAC and GEM group, caspase3 protein levels were higher than in either group alone or the control group. The transcriptome sequence, KEGG, and PPI analysis showed that the differential genes after combined treatment were mainly enriched in signal pathways related to cell proliferation, adhesion, and migration compared with using alone and control groups. Based on the sequencing results, we further investigated the role of DAC and GEM in ferroptosis-related signaling molecules using RT-PCR and Western blot techniques. RT-PCR and western blotting showed that the expression levels of HMOX1 and EBV cleavage gene BRLF1 were higher in the group with combined DAC and GEM than in the group alone and the control group, while the protein and mRNA expression levels of SLC7A11 were lower than the others. In addition, the GPX4 protein expression level in the combination group was lower than in the drug-alone and control groups. In addition, the combination treatment increased the ROS level of NK92MI cells.

**Conclusion:** Our current findings suggested that decitabine had an inhibitory effect on the proliferation of NK92MI cells when co-treated with gemcitabine. This combination may increase the expression of ferroptosis-related signaling molecules, thus inhibiting the proliferation of NK92MI cells. It also promoted apoptosis in NK/T cell lymphoma. For patients with NK/T cell lymphoma, this novel combination may provide clinical benefits.

## 1 Introduction

Since Epstein-Barr virus (EBV) has been detected in Burkitt’s lymphoma (BL) in Africa, its oncogenicity was confirmed as it could transform resting B cells into long-living cells *in vitro* (Pope et al., 1968). The virus is associated with proliferative lesions and malignant lymphomas of B, T, and NK cell origin in lymphoid tissue (Shannon-Lowe et al., 2017). Patients with EBV-positive lymphoma have a more dismal outcome and are usually treated in the same manner as EBV-negative lymphomas (Oyama et al., 2007). Patients with NK/T cell lymphoma (NKTCL) are ineffective to anthracycline-based chemotherapy due to high expression of multidrug-resistant P-glycoprotein (Yamaguchi et al., 1995). It is usually treated with a combination of radiotherapy and chemotherapy. Patients with stage I and II NKTCL are usually treated with radiotherapy, but the systemic recurrence rate is surprisingly high (Tse and Kwong, 2015). Asparaginase has a good survival rate for limited-stage NKTCL, with ORR and CR exceeding 75%, but not for advanced-stage NKTCL. Combination chemotherapy regimens including GELOX (pegaspargase, gemcitabine, oxaliplatin) (Wang et al., 2013), p-GEMOX (pegaspargase, gemcitabine, oxaliplatin) (Xia et al., 2021), MESA (methotrexate, etoposide, dexamethasone, and pegaspargase) (Xu et al., 2017), SMILE (dexamethasone, methotrexate, ifosfamide, L-asparaginase, and etoposide) (Kwong et al., 2012), DICE-L-sp (pegaspargase, gemcitabine, oxaliplatin) (Dong et al., 2016), LVP(pegaspargase, gemcitabine, oxaliplatin) (Jiang et al., 2012) all showed good clinical performance with 5-year PFS ranging from 64% to 83%. In contrast, L-asparaginase exhibits high immunogenicity and is less widely used, and its substitute, pegaspargase, is also associated with hyperlipidemia and fatal pancreatitis. Some novel targeted drugs have been investigated to overcome conventional therapies’ limitations. Anti-PD-1 monoclonal antibodies activate depleted T cells and promote immune effects for anti-tumor purposes. Among the other agents being tested in clinical trials is lenalidomide, which can stimulate angiogenesis and immunomodulate the immune system (Jones et al., 2016; Kishimoto et al., 2019). In addition, infusion of cytotoxic T lymphoma with specific antigenic specificity can re-establish immunity to EBV, thus targeting malignant cells or already infected cells (Heslop et al., 2010). However, limitations such as high cost of targeted drugs, lack of prospective studies, safety issue of biological therapies, and complexity of preparing biological materials hamper clinical applications of these methods. Hence, a combination of old medications seems to be cost-effective and practical.

Decitabine (5-Aza-2′-deoxycytidine, DAC) is a natural adenosine analog of 2′-deoxycytidine, which inhibits tumor cell proliferation by inhibiting DNA methyltransferase and reducing DNA methylation. DAC activates hypermethylated silenced genes at low doses and exerts cytotoxic effects at high doses. DAC is available for various hematologic tumors (Seelan et al., 2018; Gao et al., 2020; Patel et al., 2021; Wu et al., 2021). Furthermore, DAC has been shown to be highly effective in treating malignant lymphomas (Dalton et al., 2020; Liu et al., 2021; Zhang et al., 2021). Low-dose DAC has been shown to initiate LMP1 demethylation in EBV-positive Burkitt lymphoma. (Dalton et al., 2020). In conjunction with adoptive T-cell therapy, DAC has improved the prognosis for patients with EBV-positive Hodgkin lymphoma (Cruz et al., 2011).

Gemcitabine ((2′,2′-difluoro 2′-deoxycytidine, GEM) is a pyrimidine analog named after its structure. GEM has shown apparent efficacy in various malignancies (Mini et al., 2006; Gesto et al., 2012; Abdel-Rahman et al., 2018). Studies have shown that when combined with other cytotoxic drugs, GEM has good efficacy and is less toxic in treating EBV-associated lymphomas (Xia et al., 2021; Xie et al., 2021; Zhang et al., 2022). Additionally, GEM induces lytic EBV in nasopharyngeal carcinoma tumor cells, enabling them to be treated with antiviral therapy.

In this study, EBV-positive lymphoma-derived NK92MI cells were used to assess the synergistic effects and mechanisms of the DAC combination of the GEM.

## 2 Materials and methods

### 2.1 Cell line

NK-92MI cells were obtained from Procell Life Science&Technology Co, Ltd. They were cultures in αMEM(Gibco, Cat#41061029) medium supported with 12.5% heat-inactivated FBS(Hyclone, Cat#SH30088.03HI), 12.5% heat-inactivated horse serum (Gibco, Cat#26050088), 0.2 mM inositol (Solarbio, Cat#I8050), 0.1 mM 2-mercaptoethanol (Solarbio, Cat#M8210), and 0.02 mM folic acids (Solarbio, Cat#P1400). All media were supplemented with 100 U/mL penicillin and 100 mg/mL streptomycin (Solarbio, Cat#P1400). Cells were cultivated at 37 °C in a humidified atmosphere of 5% CO_2_ in the air.

### 2.2 Cell proliferation assay

For the growth inhibition assay, NK92MI cells were grown in 96-well plates at a density of 4 × 10^5^/mL. After the cell growth was stabilized, treated with different doses of decitabine (DAC) or gemcitabine (GEM) for 48 h, then Cell Counting Kit-8 (CCK-8, Dojindo, Tokyo, Japan) was added to each well and was incubated at 37 °C in a humidified atmosphere of 5% CO_2_ in the air for 4 h to assess the cell viability. An enzyme-labeled instrument measured absorbance at a wavelength of 450 nm. IC10, IC20, and IC30 values for two drugs were calculated using Prism six software. In order to investigate the synergistic Effect of two drugs, NK92MI cells were treated with IC10, IC20, and IC30 of DAC and IC10, IC20, and IC30 of GEM alone and in cross-pairing, respectively. The proportion of live cells was measured by CCK-8 regent. The effects of combinations were estimated using the Compusyn software, which was developed based on the median-effect method created by Chou and Talalay.

### 2.3 Apoptosis assay

For this assay, the cells were seeded in 6-well plates at a density of 4 × 10^5^/mL, which were treated with the best effective combinations. After 48 h of treatment, cells were collected and washed with PBS. Then, the cells were stained with AnnexinV Alexa Fluor488/PI(Solarbio, Cat#CA1020) according to the manufacturer’s protocol. Next, the stained cells were analyzed using a FACS Canto II flow cytometer (BD Bioscience, United States), and the data were analyzed using FlowJo version software (FlowJo LLC, United States).

### 2.4 Cell cycle assay

The cells were plated in 6-well plates at a density of 4 × 10^5^/mL. They were treated with the best effective combinations to analyze the cell cycle, which was analyzed by measuring DNA content using a flow cytometer (FACS Canto, BD Bioscience, CA). In the following step, the cells were collected and washed with PBS, and then they were fixed overnight in ice-cold 70% ethanol at −20°C. Next, the cells were resuspended in PBS, incubated with 100 µL RNase at 37 °C for 30 min, and stained with 400 µL PI(Solarbio, Cat#CA1510) at 4 °C for 30 min in the dark prior before flow cytometer analysis.

### 2.5 RNA-seq

After drug treatments, the Total RNA of each group was extracted by TRIzol reagent. Then, RNA sequencing was performed, and its data was analyzed by the Annaroad Gene Technology (Shanghai, China) Co., Ltd.

### 2.6 RT PCR

The total RNA of the cells was extracted using TRIzol, and RNA was eluted with RNase-free water and quantified at an absorbance of 260/280 nm. Then cDNA was synthesized by using HiFiScript gDNA Removal cDNA Synthesis Kit (Cwbio, Cat#CW 2020). Real-time PCR was performed in an optional 96-well plate with ABI7500 system and a commercial MagicSYBR mixture kit (Cwbio, Cat#CW3008M), according to the manufacturer’s instructions. GAPDH was used as an endogenous control.

### 2.7 Western blot

The cells were collected and lysed on ice by RIPA (Solarbio, Cat#R0010) lysis buffer containing 1% PMSF. Protein concentration was determined by BCA protein assay kit (Beyotime, Cat#ST2222). 30 μg of total lysate protein sample was separated by SDS-PAGE (Shanghai Epizyme Biomedical Technology Co., Ltd, Cat#PG114) and transferred onto the NC membrane (Merck, Cat#HATF02500). Then membranes were incubated overnight at 4 °C with primary antibodies. Membranes were washed with Western washing buffer triplicates at room temperature and then incubated with secondary antibody for 1 h at room temperature. Membranes were rewashed with Western washing buffer triplicates at room temperature, and results were acquired using the Gel Logic 1,500 imaging system. *GAPDH* was used as the loading control.

### 2.8 Reactive oxygen species assay

The collected cell suspension was centrifuged at 250 g for 5 min and washed twice with PBS. Dilute DCFH-DA with serum-free culture medium at 1:1,000 to a final concentration of 10 μmol/L, add 1 mL of DCFH-DA (Beyotime, Cat#S0033M) dilution to each tube, and incubate in a 37°C cell culture incubator for 20 min. Next, the cells were washed three times with a serum-free cell culture medium to remove DCFH-DA that did not enter the cells fully. After loading the probes in the positive control group, dilute ROS-up with the serum-free medium at a ratio of 1:1,000 to make ROS-up dilution, add 1 mL of ROS-up dilution to the positive control, and incubate in a cell culture box at 37°C for 30 min. Cells were washed three times with serum cell culture medium. After washing once with pre-cooled PBS, the cell pellet was resuspended with 1 mL of PBS and immediately detected using a FACSCanto II flow cytometer according to the manufacturer’s instructions. Flow cytometry results were analyzed with the software FlowJo.

### 2.9 Statistical analysis

Numerical data were expressed as mean ± SD, and statistical analyses were performed using unpaired *t*-test by SPSS 26.0 software. P < 0.05 was considered to be statistically significant.

## 3 Results

### 3.1 The combination of DAC and GEM showed synergistic inhibition of cell viability on NK92MI cells

**FIGURE 1:**
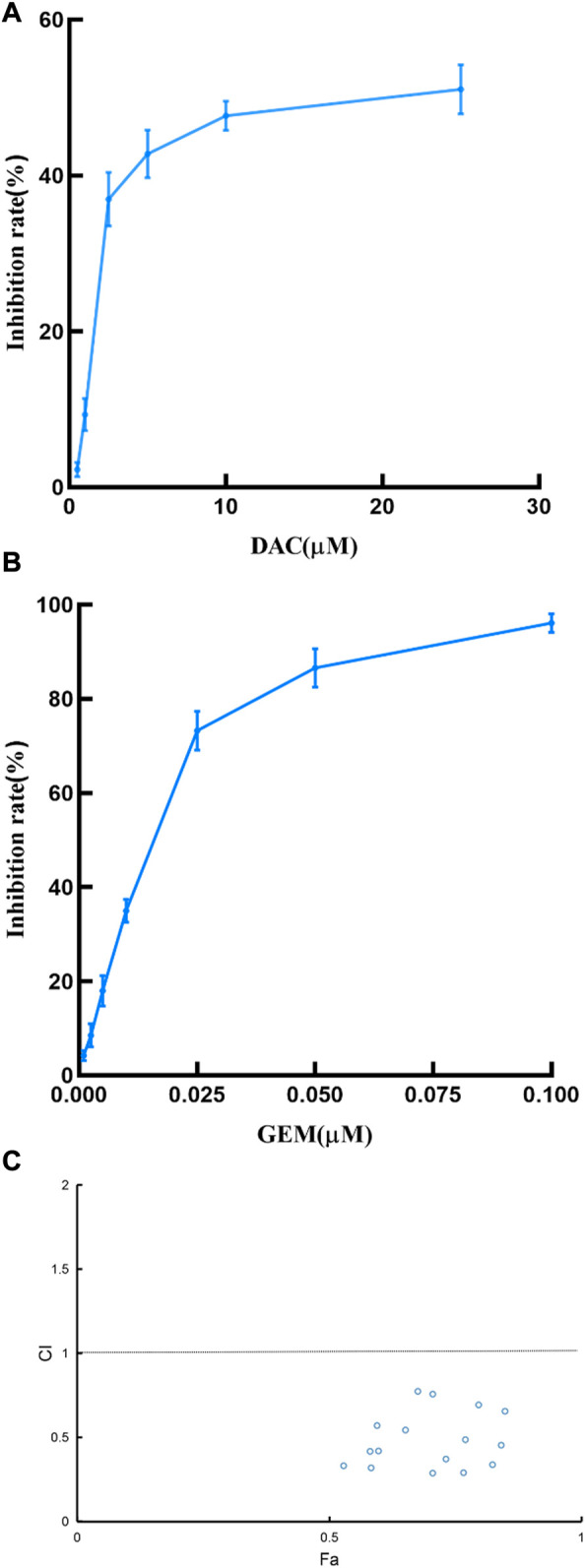
Effects of DAC and GEM on NK92MI cell viability and confirmation of the best combination of DAC and GEM. **(A)** Effects of DAC on cell growth in NK92MI cell lines. **(B)** Effects of GEM on cell growth in NK92MI cell lines. **(C)** CI plots of DAC and GEM combinations in NK92MI cells. The combination index was estimated using Compusyn software. CI < 1 indicates synergy; CI = 1 indicates an additive effect; CI > 1 indicates antagonism.

We first determined the inhibition of cell viability of DAC and GEM on the EBV-positive lymphoma cell line NK92MI cells separately. NK92MI cells were treated with DAC and GEM gradient concentrations for 48 h. Both DAC and GEM exhibited dose-dependent proliferation inhibitory effects on NK92MI cells (Figures 1A, B). Next, the IC10, IC20, and IC30 values were calculated for DAC and GEM (Table 1). We performed a 48-h cross-combination of DAC and GEM in NK92MI cells with IC10, IC20, and IC30. The combination index (CI) values were under one in all groups (Table 2; Figure 1C). As expected, the combination caused a significant reduction in cell growth compared to drugs alone. For further experiments, we selected the pairing with the lowest CI value, representing the highest synergistic efficiency (Section 3.5).

**TABLE 1:** IC10, 1C20, and IC30 of DAC and GEM in NK92MI cells.

Inhibition rate	DAC (µM)	GEM (µM)
IC10	1.603	0.005
IC20	2.100	0.007
IC30	4.046	0.010

**TABLE 2:** Proliferation inhibition rate or combination index of NK92MI cells treated with different concentrations of DAC and GEM.

DAC (µM)	GEM(µM)	Inhibition rate (%)	Combination index
1.603	0.005	54.33 ± 1.06	0.379 ± 0.039
1.603	0.007	57.16 ± 2.44	0.474 ± 0.041
1.603	0.010	58.80 ± 2.12	0.631 ± 0.045
2.100	0.005	60.07 ± 1.53	0.361 ± 0.039
2.100	0.007	61.13 ± 3.19	0.457 ± 0.033
2.100	0.010	64.90 ± 1.20	0.592 ± 0.036
4.046	0.005	72.09 ± 1.43	0.322 ± 0.030
4.046	0.007	74.74 ± 2.76	0.389 ± 0.033
4.046	0.010	76.99 ± 1.92	0.510 ± 0.046

### 3.2 The combination of DAC and GEM significantly promotes apoptosis in NK92MI cells

**FIGURE 2:**
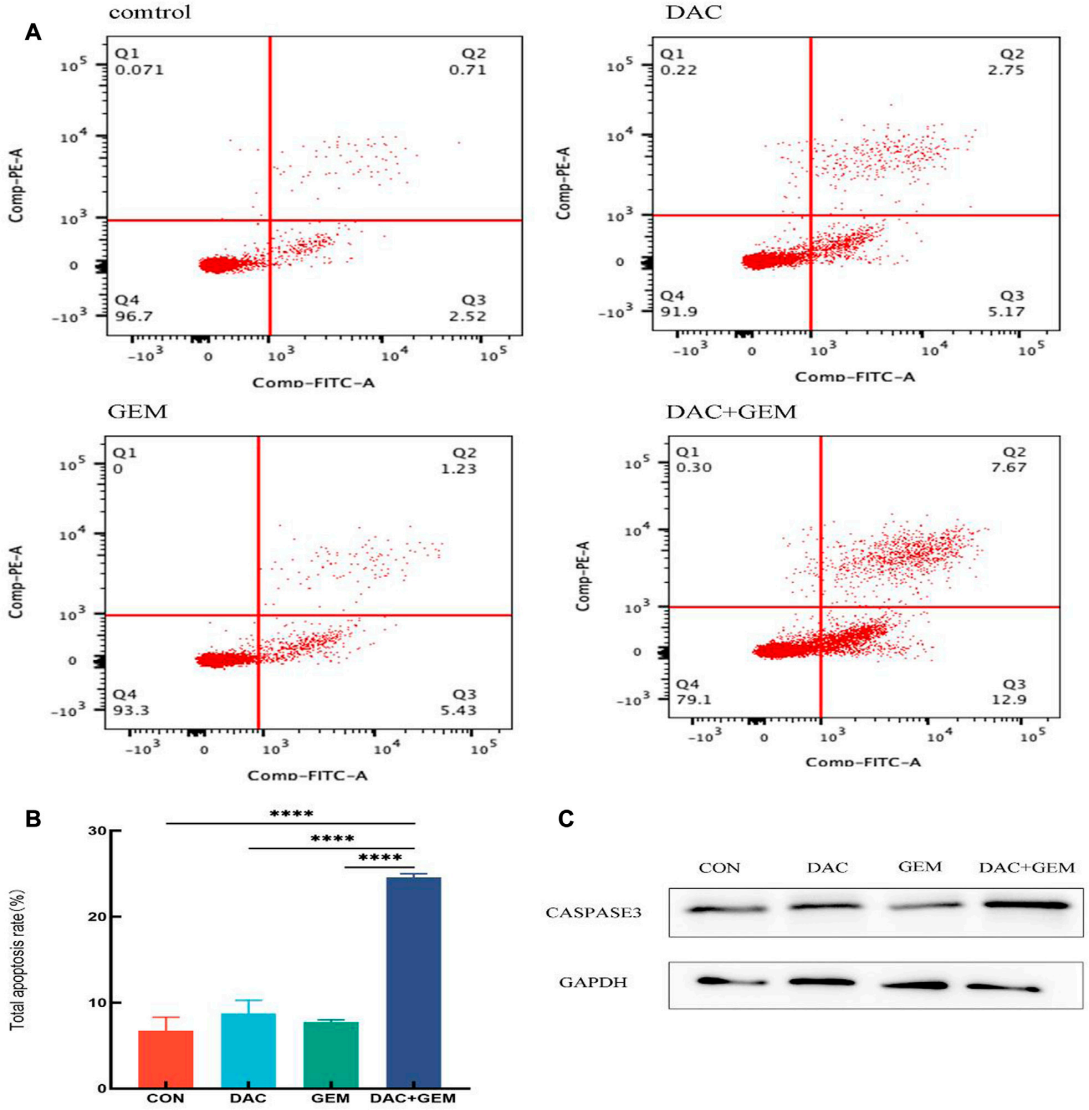
The combination of DAC with 4.046 µM and GEM with 0.005 µM significantly promotes apoptosis in NK92MI cells. **(A, B)** Cell apoptosis assay in NK92MI cells treated with the indicated dose of DAC and GEM alone or in combination. **(C)** After treating with the indicated dose of DAC and GEM alone or in combination, Caspase-3 protein expression was immunoblotted in NK92MI cells. *p < 0.05, **p < 0.01, ***p < 0.001 versus control group and alone group.

We performed a flow cytometric analysis on the intervened cells to confirm whether DAC and GEM promote apoptosis. We incubated the cells for 48 h under the following conditions: 4.046 µM DAC, 0.005 µM GEM, 4.046 µM DAC+ 0.005 µM GEM. The apoptosis rate of the control group was 6.76% ± 1.54%, the DAC group was 8.74% ± 1.53%, the GEM group was 7.72% ± 0.28%, and the combined was 24.57% ± 0.42%. The apoptosis rate was increased in the group DAC + GEM than DAC group (*p* < 0.05) or GEM group (*p* < 0.05) (Figures 2A, B). In addition, the western blot demonstrated that caspase3, an apoptosis-associated marker, was increased by DAC, GEM, and DAC-GEM. A significant increase in expression was observed in the combined group (Figure 2C). These results show that DAC can synergistically induce apoptosis in EBV-positive lymphoma NK92MI cells with GEM.

### 3.3 The combination of DAC and GEM induces arrest in the S phase of EBV-positive lymphoma cells

**FIGURE 3:**
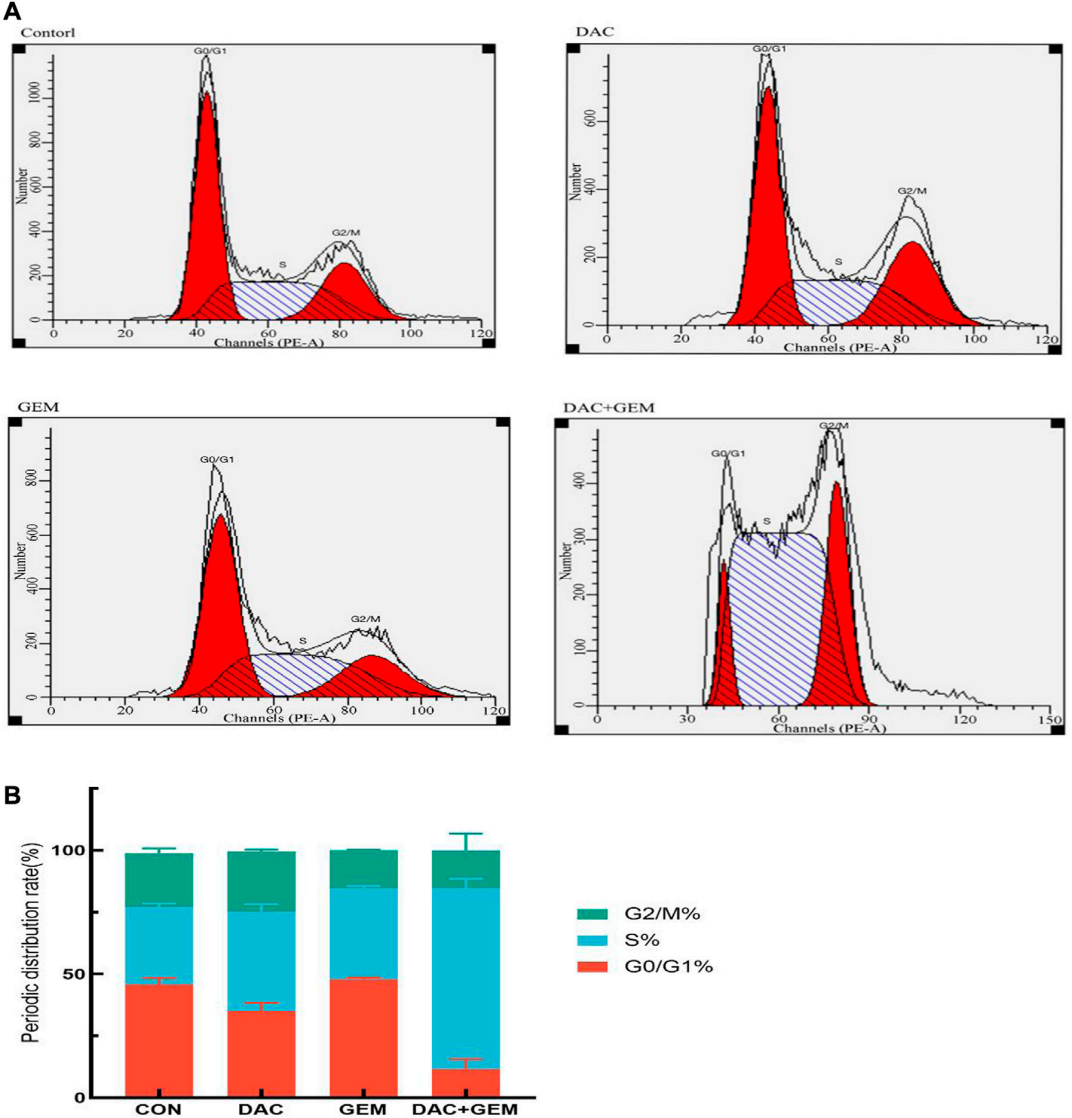
The cell cycle distribution of NK92MI cells after 48-h exposure to DAC and GEM. **(A, B)** The proportion of NK92MI cells in the S phase in the combination of DAC with 4.046 µM and GEM with 0.005 µM was significantly higher.

We treated NK92MI cells in the same group as before for 48 h and then measured the cell cycle by flow cytometry. We found that the proportion of cells in the S phase in the combined DAC and GEM group (72.82 + 3.09)% was significantly higher than that in the DAC (40.35 + 2.93)% and GEM (36.86 + 0.84%) alone groups. Compared with the control group (31.40 + 1.48%), the proportion of S phase cells increased regardless of whether the drug was used alone or in combination (Figures 3A, B). The results showed that DAC plus GEM could block the cell cycle progression of EBV-positive lymphoma cells NK92MI in the S phase and reduce the cells in the proliferative phase.

### 3.4 Combination of DAC and GEM induced transcriptome level changes

**FIGURE 4:**
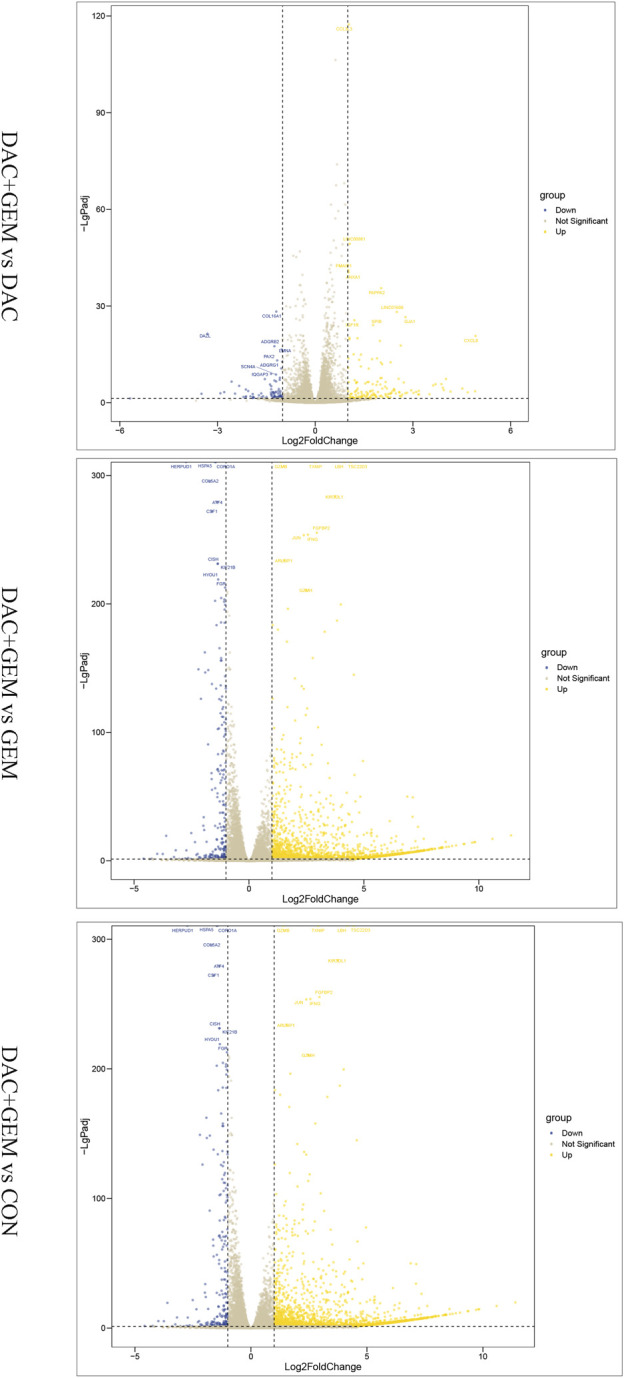
Combination of DAC and GEM induced transcriptome level changes. After treatment with the indicated dose of DAC and GEM alone or in combination, a volcano plot of differentially expressed genes in the combination group was compared with each single drug group or control group in NK92MI cells.

To assess gene expression changes mediated by different treatments, we performed transcriptome sequencing on the control group, the DAC group, the GEM group, and DAC plus GEM groups using RNA-seq technology. Compared with the control group, NK92MI cells treated with DAC and GEM showed 2,382 gene expression changes, of which 2,142 were upregulated and 240 were downregulated. There were 235 gene expression changes between the DAC group and the combination group, of which 149 genes were upregulated and 86 genes were down-regulated. A total of 2,204 genes were induced by DAC plus GEM compared with the GEM group, with 2,056 genes being upregulated and 148 genes being downregulated. The results indicate that DAC was the primary mediator of the changes in gene expression profiles caused by the use of both drugs (Figure 4).

**FIGURE 5:**
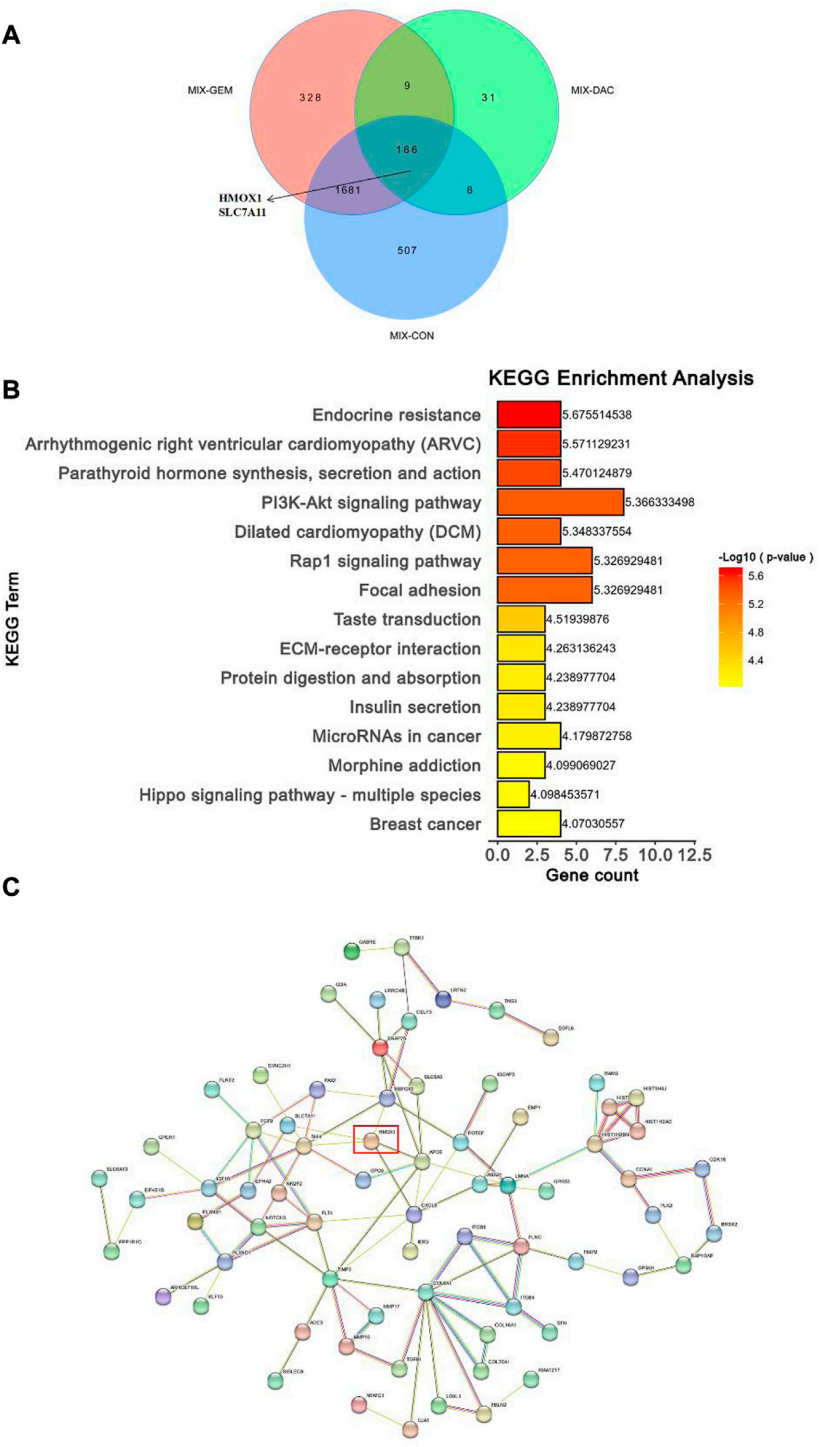
Combination of DAC and GEM induced transcriptome level changes. **(A)** The Venn diagrams showed the DEGs in the combination group when compared with each single drug group and control group in NK92MI cells. **(B)** The primary signal transduction pathways of DEGs in the combination group compared with each single drug group and control group in NK92MI cells. **(C)** The protein-protein interaction network topology analysis of DEGs in the combination group compared with each single drug group and control group in NK92MI cells.

As a next step, we utilized Venn diagrams to visualize the number of differentially expressed genes (DEGs) in the combined group compared with the single and control groups. Based on the analysis results, 186 differentially expressed genes were significantly altered when the DAC and GEM were connected (Figure 5A). Then we performed pathway enrichment analyses comparing DEGs between DAC, GEM, and DAC plus GEM treated cells using the well-annotated reactome and Kyoto Encyclopedia of Genes and Genomes (KEGG) databases. Compared to the other three groups, the DEGs were primarily enriched in signaling pathways related to cell proliferation, adhesion, and migration in the combination treatment (Figure 5B). Such as Focal adhesion, PI3K Akt signaling pathway, and Rap1 signaling pathway. Using PPI network analysis, we investigated the interaction between DEGs by analyzing their interactions. The networks formed by DEGs mainly regulate metabolic pathways and cell proliferation and migration pathways (Figure 5C). For further investigation, we selected molecules with high node expression and high relative expressions in 186 DEGs for validation, such as HMOX1 and SLC7A11.

### 3.5 The possible mechanism of the combined application of DAC and GEM to inhibit cell viability

**FIGURE 6:**
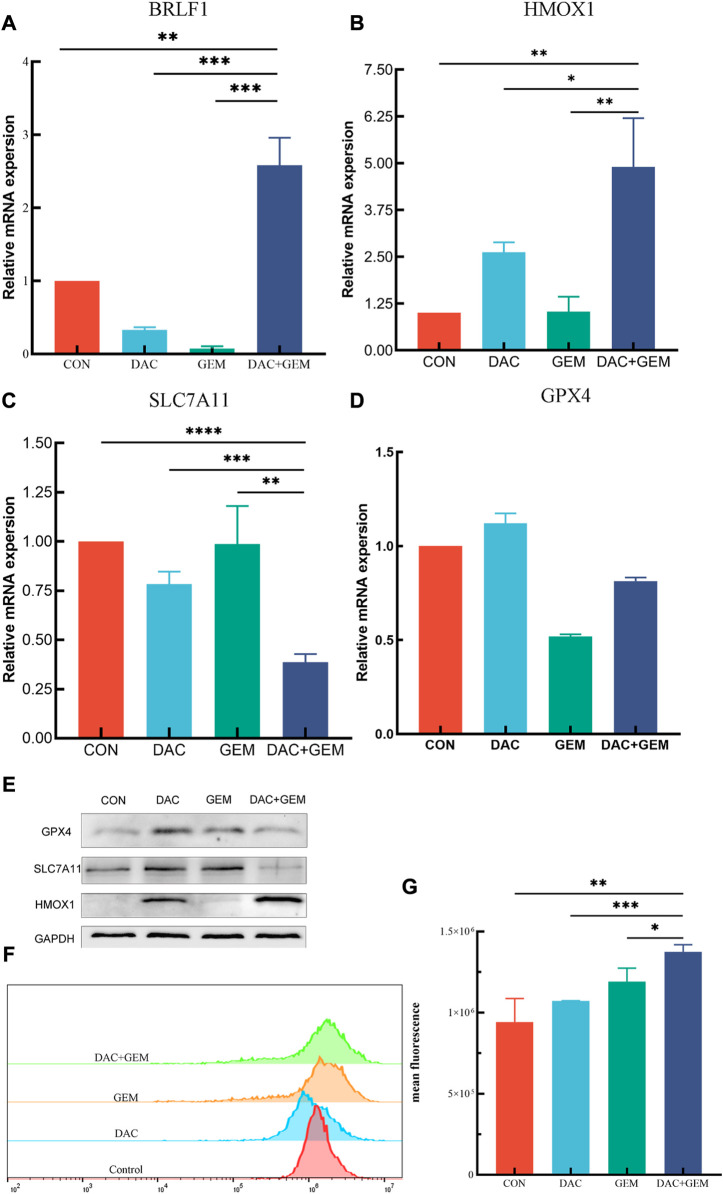
Combined treatment of DAC and GEM assists ferroptosis. **(A)** After treating with the indicated dose of DAC and GEM alone or in combination, BRLF1 mRNA expression was quantified by RT-PCR in NK92MI cells. **(B)** After treating with the indicated dose of DAC and GEM alone or in combination, HMOX1 mRNA expression was quantified by RT-PCR in NK92MI cells. **(C)** After treating with the indicated dose of DAC and GEM alone or in combination, SAL7A11 mRNA expression was quantified by RT-PCR in NK92MI cells. **(D)** After treating with the indicated dose of DAC and GEM alone or in combination, GPX4 mRNA expression was quantified by RT-PCR in NK92MI cells. **(E)** After treatment with the indicated dose of DAC and GEM alone or in combination, HMOX1, SAL7A11, and GPX4 protein expression were immunoblotted in NK92MI cells. **(F, G)** The effects of DAC and GEM on regulating ROS level in NK92MI cells. *p < 0.05, **p < 0.01, ***p < 0.001 versus control group and alone group.

We also chose *BRLF1* as an additional observation in order to judge whether the EBV lytic state should be activated. After treatment with the indicated dose of DAC and GEM alone or in combination, *BRLF1* mRNA expression was by RT-PCR in NK92MI cells. The combination of DAC and GEM induced the expression of *BRLF1*. This combination may break the viral latency (Figure 6A).

RT-PCR and Western Blot experiments were performed on HMOX1 and SLC7A11 to study the mechanism of action of DAC and GEM combined to inhibit cell proliferation. The results showed that the combined treatment of DAC and GEM with NK92MI cells induced the expression of HMOX1 at the mRNA level (*p* < 0.05) and protein level and inhibited the expression of SLC7A11 at the mRNA level (*p* < 0.05) and protein level (Figures 6B, C, E). Based on these results, it is possible that DAC + GEM may influence the changes in ferroptosis-related molecules. GPX4 is crucial in regulating ferroptosis, and SLC7A11 serves as its upstream mediator. To explore whether the proliferation inhibition induced by the combined application of DAC and GEM is associated with ferroptosis-related genes in NK92MI cells, we further investigated the protein and mRNA levels of GPX4. There was no significant difference in GPX4 mRNA levels between the single and control groups (Figure 6D); however, the protein expression of GPX4 was decreased (Figure 6E). The primers used in the study are listed in Table 3.

**TABLE 3:** Primer sequences used for RT-PCR detection.

Primer name	Sequence (5′–3′)
GAPDH-F	TGA​CAA​CTT​TGG​TAT​CGT​GGA​AGG​AC
GAPDH-R	GTG​TCG​CTG​TTG​AAG​TCA​GAG​GAG
BRLF-F	GAG​TCC​ATG​ACA​GAG​GAT​TTG​A
BRLF-R	GCA​GCA​GAC​ATT​CAT​CAT​TTA​GA
HMOX1-F	AAG​ACT​GCG​TTC​CTG​CTC​AAC
HMOX1-R	AAA​GCC​CTA​CAG​CAA​CTG​TCG
SLC7A11-F	ACC​TTT​TCT​GAG​CGG​CTA​CT
SLC7A11-R	CCC​TCT​CGA​GAC​GCA​ACA​TA
GPX4-F	ATA​CGC​TGA​GTG​TGG​TTT​GC
GPX4-R	CTT​CAT​CCA​CTT​CCA​CAG​CG

### 3.6 The combined application of DAC and GEM increases the ROS level of NK92MI cells

As a result of an increase in reactive oxygen species (ROS) related to iron, lipid peroxidation occurs, which impairs cellular function and ultimately results in ferroptosis. Therefore, we examined ROS levels in four groups using flow cytometry. The mean fluorescence intensities of each group were: the control group was 7.9 × 10^6^, the DAC group was 10.7 × 10^6^, the GEM group was 12.7 × 10^6^, and the combination group was 14.1 × 10^6^ (Figures 6F, G). The results showed that ROS levels in NK92MI cells were significantly elevated after the combined treatment of DAC and GEM(*p* < 0.05).

## 4 Discussion

EBV is a “kiss virus” that spreads mainly through the respiratory tract in humans. About 95% of the population have been infected in childhood and carried it in a latent state for life. In immunocompromised patients, EBV activates B cells and leads to the malignant proliferation of B cells to trigger monoclonal or polyclonal lymphoproliferative disorders or EBV-related malignancies. Studies have shown that LMP1 and LMP2 can be detected in 30%–45% of human lymphoma cells (Ramos et al., 2016). Previous clinical trials found that lymphoma patients with EBV usually have a high probability of extranodal involvement, advanced stage, older age, and higher risk (Beltran et al., 2011; Ok et al., 2014; Witte et al., 2020).

NK/T-cell lymphoma is a non-Hodgkin’s lymphoma with prominent geographical characteristics. It is more common in Asians and Latin Americans to develop NK/T-cell lymphoma, especially in patients with EBV positivity. The disease is characterized by a high degree of malignancy, extreme aggressiveness, rapid progression, and high recurrence rates. At present, there is no standard treatment for NK/T cell lymphoma, and asparaginase and gemcitabine are usually used as primary chemotherapy agents. However, the prognosis of this disease is inferior due to the emergence of drug resistance in many patients. Therefore therapeutic regimens targeting both the EBV virus and lymphoma cells are needed.

DAC is a highly efficient inhibitor of DNA methyltransferase. DAC can induce self-renewal of normal hematopoietic stem cells, which has excellent potential for treating hematological diseases (Pinto et al., 1984; Hu et al., 2010). Zhang et al. reported a phase 1/2 and biomarker study on the Effect of DAC combination with R-CHOP in patients with diffuse large B cell lymphoma; 76.6% of patients achieved complete remission, and 12.2% of patients achieved partial remission (Zhang et al., 2021). GEM is an antimetabolite drug targeting specific stages of the cell cycle. It promotes the death of tumor cells with cytotoxicity and is widely used in clinical practice. Chen et al. (2021) demonstrated that Gem and thymosin alpha one combined suppressed NNKTL progression *in vivo* and *in vitro*. A therapeutic strategy for NNKTL could potentially be used in clinical practice due to their study. According to research reports, GEM combined with cisplatin and dexamethasone has a good effect in treating refractory non-Hodgkin lymphoma, with an overall response rate of 56.4% and an overall response rate of 72.8% in the treatment of refractory Hodgkin lymphoma (Mi et al., 2020). The above studies show that GEM can safely and effectively improve anti-tumor efficiency when combined with other drugs. In addition, GEM can induce EBV from a latent state to a lytic state, improving tumor cells’ sensitivity to antiviral therapy (Feng et al., 2004; Wildeman et al., 2012; Chen et al., 2021).

Few studies have combined low doses of DAC and GEM. Clouser et al. found that low doses potently inhibited HIV-1 replication *in vitro* and can inhibit the progression of the disease *in vivo* (Clouser et al., 2012). Valdez et al. designed a safe and effective pretreatment protocol for hematopoietic stem cell transplantation. Combining busulfan, melphalan, GEM and DAC inhibited lymphoma cell growth. Additionally, a recent study demonstrated that the combination of DAC and GEM inhibited osteosarcoma cell proliferation and retarded tumor growth (Gutierrez et al., 2022). We studied DAC and GEM’s *in vitro* antiproliferative effects on NK92MI. We demonstrated that DAC and GEM single-drugs are effective against the growth of NK92MI cells in dose-dependent manners. The combined drug effect was better than the single. In tumor cells, ROS accumulation is a double-edged sword; a slight increase in ROS promotes tumor cell proliferation, while an excessive increase can lead to cancer cell death. It is important to note that apoptosis is one of the most common methods of cell death. Apoptosis is divided into intrinsic, extrinsic, and perforin/granzyme pathways (Romero et al., 2015). Induced by intracellular stress, apoptosis is primarily initiated *via* the intrinsic pathway. The mitochondrial outer membrane is altered by ROS accumulation, releasing cytochrome C and activating caspase-9 in the apoptotic body. This causes caspase-3 to be activated, which is the ultimate executor of apoptosis (Ashkenazi and Dixit, 1998). Our study first demonstrated that the combination of DAC and GEM could effectively inhibit the proliferation of NK92MI cells, increase ROS accumulation, increase caspase3 protein level, promote cell apoptosis, and induce an S phase arrest. These results showed that the apoptotic process induced the combination by upregulating caspase3. However, the effects of DAC plus GEM on anti-proliferation may not only depend on apoptosis.

After initial infection, the virus establishes latency predominantly in B cells and cannot be cleared up. A persistent state of latent infection is closely associated with malignancies of human epithelial cell origin and lymphocytic origin. However, the viral latency is disrupted if external stimuli infect cells. And then, the virus enters a lytic state (Munz, 2019; Rosemarie and Sugden, 2020; Munz, 2021). Our study found that the combination group increased *BRLF1* expression in NK92MI cells compared to the single group. As is well known, EBV lytic activation is closely correlated with the expression of two EBV immediate early genes *BRLF1*, and *BZLF1.* Based on the study’s results, it appears that the combination of DAC and GEM may induce replicative lysis of EBV in the NK92MI cell line.

Following this, we investigated other mechanisms of DAC and GEM’s antiproliferative effects on NK92MI. In this experiment, we investigated the gene expression levels between the single and combination groups after 48 h of nk92mi cell treatment using whole gene transcriptome sequencing. A total of 186 differential genes were screened when the combined group was compared with the single and control groups. HMOX1 and SLC7A11 were found to be responsive to the combinations of DAC and GEM by KEGG enrichment analysis and PPI network analysis. Compared to the single and control groups, the combination group increased the expression of HMOX1 and decreased the expression of SLC7A11 at mRNA and protein levels. HMOX1 is a rate-limiting enzyme in the metabolism of the iron porphyrin compound heme catabolism, which breaks down heme to carbon monoxide, biliverdin, and Fe^2+^. HMOX1 expression is mainly induced by its heam, iron, oxidative stress, and inflammation (Chang et al., 2015). HMOX1 is vital in maintaining iron homeostasis and protecting cells from oxidative damage. It has been shown that adequate levels of HMOX1 can act as antioxidants and prevent cell oxidative damage (Kim et al., 2012). Chang et al. (2015) indicated that treatment with DNA methylation transferase inhibitors significantly increased the expression of HMOX1 mRNA (Sung et al., 2016). In the present study, we observed an increased expression of HMOX1 in the DAC-alone group compared to the control group. The expression of HMOX1 was significantly increased in the combined DAC and GEM group compared with the control group and the drug-alone group. This phenomenon may be caused by DAC’s inhibitory effect on methylation, which was amplified by the combination with GEM. However, overexpression of HMOX1 and Fe^2+^ accumulation and ROS accumulation exert a role in inducing cellular ferroptosis. In ferroptosis, there is an imbalance of intracellular iron and depletion of glutathione, resulting in the accumulation of ROS and lipid peroxidation.

Although ROS is not a specific indicator of ferroptosis, ROS plays an important role in ferroptosis. Kong et al. (2019) observed an increase in Fe and an increase in ROS after the artesunate treatment of hepatic stellate cells in a study of liver fibrosis in mice treated with artesunate. In a study on paraquat + maneb-induced dopaminergic neurodegeneration, NADPH oxidase was found to be involved in ferroptosis. When NOX was inhibited with NADPH oxidase, cellular GSH and GPX4 levels were restored, and ferroptosis was reduced (Hou et al., 2019). DAC caused ROS accumulation in leukemia cells but not in solid tumor cells, thereby promoting leukemic cell death (Fandy et al., 2014; Jain et al., 2017). Our study also showed that the combination induced an increase in ROS levels. It is well known that SLC7A11 and GPX4 are critical molecules in ferroptosis. GPX4 is the core enzyme regulating the glutathione system of the endogenous antioxidant system, and inhibition of GPX4 activity leads to ROS overload, disrupting cell membranes and thus inducing ferroptosis (Dixon et al., 2012). The efficiency of cysteine transport directly affects glutathione synthesis, and its main depends mainly on the cystine/glutamate reverse transporter protein in the cell membrane. SLC7A11, an essential part of the cystine/glutamate reverse transporter protein, is a negative ferroptosis regulator. SLC7A11 downregulation affects glutathione synthesis’s inhibition, leading to GPX4 downregulation, cellular ROS accumulation, and ultimately oxidative damage and ferroptosis.

Interestingly, this study’s protein level of GPX4 was down-regulated, but the mRNA level did not change significantly. The combination caused the downregulation of GPX4 and SLC7A11, leading to an overload of ROS, which induced the expression of HMOX1. The recent discovery that early transformed stages of EBV and Burkitt’s lymphoma cells trigger ferroptosis suggests that ferroptosis may be therapeutically beneficial in treating EBV-positive lymphomas (Burton et al., 2022). This may be another reason why this combination inhibited cell growth proliferation.

In conclusion, the combination of DAC and GEM has synergistic effects for cell viability inhibition in NK92MI cells through apoptosis and ferroptosis. Although the combination of DAC and GEM has mediated many forms of anti-proliferation, more research is needed to determine which form is critical. Our study has some limitations. First of all, our study mainly discussed the inhibitory effect of DAC + GEM on the proliferation of NK92MI cells *in vitro* and did not observe the effect *in vivo*. The construction of a xenograft mouse model is of great significance for exploring the inhibition of tumor cells by DAC + GEM *in vivo*. In particular, the construction of PDX models can more truly reflect the original characteristics of tumors and more accurately reflect the drug sensitivity and tolerance of tumor patients. Secondly, although this study suggests that DAC + GEM may inhibit the proliferation of NK/T-cell lymphoma cells through apoptosis or ferroptosis, we still need more evidence to prove whether these two mechanisms play a major inhibitory role or play a joint inhibitory role. This study demonstrates that the combination of DAC and GEM is promising for inhibiting NK/T cell lymphoma cells and provides new ideas for the clinical treatment of NK/T cell lymphoma.

## Data Availability

The raw data generated in this study have been deposited in the SRA database (https://www.ncbi.nlm.nih.gov/sra), under the accession numbers SRR23620573, SRR23620567, SRR23620568, SRR23620570, SRR23620575, SRR23620572, SRR23620569, SRR23620571, SRR23620574, SRR23620577, SRR23620576, SRR23620566.
